# Early Diagnosis of Glaucoma By Optical Coherence Tomography: A Systematic Review and Network Meta-analysis

**DOI:** 10.18502/jovr.v21.18852

**Published:** 2026-03-02

**Authors:** Azadeh Doozandeh, Mohammadmehdi Hatami, Zahra Khorrami, Ali Sadatmoosavi, Mohammad Farjami, Ghazale Soltani

**Affiliations:** ^1^Ophthalmic Research Center, Research Institute for Ophthalmology and Vision Science, Shahid Beheshti University of Medical Sciences, Tehran, Iran; ^2^Department of Ophthalmology, Torfeh Hospital, Shahid Beheshti University of Medical Sciences, Tehran, Iran; ^3^Ophthalmic Epidemiology Research Center, Research Institute for Ophthalmology and Vision Science, Shahid Beheshti University of Medical Sciences, Tehran, Iran; ^4^Department of Medical Library & Information Sciences, Faculty of Management and Medical Information Sciences, Kerman University of Medical Sciences, Kerman, Iran; ^5^Department of Biostatistics, Faculty of Paramedical Sciences, Shahid Beheshti Medical University, Tehran, Iran

**Keywords:** Diagnosis, Early Glaucoma, Optic Nerve Head, Optical Coherence Tomography, Retinal Ganglion Cells

## Abstract

Optical coherence tomography (OCT) diagnostic technology is increasingly being integrated into clinical practice and is evolving rapidly. Performing a meta-analysis of these advancements can provide valuable insights to clinicians and healthcare decision-makers and help identify gaps that need further research and development. We conducted a systematic review and network meta-analysis (NMA) comparing the diagnostic accuracy of three key OCT-derived parameters in identifying early glaucoma: peripapillary retinal nerve fiber layer (RNFL) thickness, macular metrics, and optic nerve head (ONH) characteristics. We systematically searched PubMed, Embase, Web of Science, and Scopus for studies published from January 2004 through March 2024, and identified 47 eligible studies. These studies comprised 12,723 eyes from 8177 participants, including patients with mild primary open-angle glaucoma, pre-perimetric glaucoma (PPG), and ocular hypertension, as well as healthy controls. Our NMA summarized the existing evidence on the diagnostic performance of these parameters. The highest diagnostic accuracies for glaucoma detection were observed for average and inferior RNFL parameters, both achieving an accuracy of 0.77 and macular GLV parameter with an accuracy of 0.76. These were followed by inferotemporal GCIPL and two ONH parameters—rim area and cup-to-disc ratio—each with an accuracy of 0.75. In conclusion, the overall diagnostic accuracy was comparable across all three categories of OCT parameters. The results also suggest the potential benefits of combining two or three of these parameters in a single report, along with the application of artificial intelligence. This approach could significantly enhance the diagnostic accuracy of OCT in detecting glaucoma at its earliest stages, ultimately improving patient outcomes through earlier intervention.

##  INTRODUCTION

Glaucoma is the leading cause of irreversible blindness worldwide. However, timely intervention can significantly improve its prognosis.^[1,2,3]^ With global population aging, the prevalence of glaucoma is expected to rise, especially among older adults.^[4]^ Early detection is essential for slowing disease progression through intraocular pressure–lowering therapies and for improving quality of life.^[5]^

Early glaucoma is typically asymptomatic, making diagnosis highly dependent on the clinician's vigilance during routine eye examinations.^[6]^ Alarmingly, many cases remain undetected even in specialized ophthalmic centers,^[6,7,8]^ largely due to limitations of conventional diagnostic tools.

Glaucoma is characterized by progressive degeneration of retinal ganglion cells (RGCs) and their axons, which form the retinal nerve fiber layer (RNFL), accompanied by structural changes in the optic nerve head (ONH) and corresponding visual field (VF) loss.^[9]^ Despite the availability of functional (e.g., perimetry) and structural (e.g., ophthalmoscopy) diagnostic modalities, distinguishing normal anatomical variability from early glaucomatous damage remains challenging.^[10]^

Optical coherence tomography (OCT) has emerged as an indispensable tool for glaucoma assessment, providing objective, quantitative measurements of retinal and ONH morphology. Among its metrics, circumpapillary RNFL thickness—a surrogate for RGC axon density—is a cornerstone of glaucoma evaluation.^[11]^ Recent advances, such as artificial intelligence (AI)-based probability maps, have enhanced the sensitivity of RNFL analysis.^[12,13]^

Beyond RNFL, spectral-domain OCT enables precise layer-specific imaging of macular structures, where approximately half of all RGCs reside.^[14]^ OCT-derived ONH parameters, such as minimum rim width (MRW), rim area (RA) and neuroretinal rim volume offer complementary information on neuroretinal integrity.

While meta-analyses have demonstrated high sensitivity and specificity of OCT parameters for moderate and advanced glaucoma,^[11]^ the diagnostic accuracy of OCT in early disease remains limited because subtle structural alterations at this stage often fall within the range of measurement variability. Furthermore, previous reviews have noted substantial heterogeneity across studies in terms of population characteristics, diagnostic criteria, and OCT device versions.^[15,16,17]^

This network meta-analysis (NMA) evaluates the diagnostic accuracy of peripapillary RNFL, macular ganglion cell complex (GCC), and ONH parameters across various OCT platforms [Supplementary Material 1]. By synthesizing evidence from diverse clinical cohorts, we aim to clarify discrepancies, identify early biomarkers, and provide practical guidance for clinical decision-making.

While pairwise meta-analysis is useful for direct comparisons, NMA enables simultaneous comparison of multiple diagnostic modalities, offering a more comprehensive understanding of relative performance.^[18]^ To our knowledge, this is the first NMA to simultaneously compare the diagnostic performance of multiple OCT parameters across different acquisition protocols within a unified analytical framework.

##  METHODS

### Study Design

This systematic review and NMA adhered to the guidelines of the Preferred Reporting Items for Systematic Reviews and Meta-Analyses (PRISMA). The PRISMA statement consists of a 27-item checklist and a four-phase flow diagram.^[19]^ Two investigators (MH and AD) were independently responsible for screening the titles, abstracts, and full-text studies and performing data extraction. Any disagreement was resolved either by a third independent reviewer (ZK) or through discussion.

### Literature Search

We conducted a comprehensive search in PubMed, Embase, Web of Science, and Scopus for studies published between January 2004 and March 2024. Search strategies were tailored for each database [Supplementary Material 2], and reference lists of included studies were also screened.

### Inclusion and Exclusion Criteria

We excluded studies on patients with moderate to advanced glaucoma. Eligible studies were required to be original research articles published between January 2004 and March 2024. They had to include participants aged 40-80 years diagnosed with ocular hypertension, pre-perimetric glaucoma (PPG), and mild primary open-angle glaucoma (POAG), all defined using consistent diagnostic standards. Ocular hypertension was defined as intraocular pressure (IOP) > 21 mmHg, normal optic disc appearance, and a normal VF with a mean deviation (MD) better than - 2 dB. PPG required IOP > 21 mmHg and clear evidence of glaucomatous optic nerve damage (e.g., neuroretinal rim thinning, excavation, or notching) in the absence of reproducible VF defects.Mild glaucoma was defined as glaucomatous optic neuropathy with a VF MD better than –6 dB, according to standard VF severity grading.^[20]^ Studies were also required to report at least one quantitative structural parameter measured using OCT, including peripapillary RNFL thickness, ganglion cell–inner plexiform layer (GCIPL) thickness, GCC thickness, Bruch's membrane opening minimum rim width (BMO-MRW), or optic disc RA.

The exclusion criteria were animal experiments, reviews, case reports and conference abstracts, studies using time-domain OCT, and studies written in languages other than English.

### Data Extraction 

Data were independently extracted by two reviewers (MH and AD) using a standard data extraction form. The following data were extracted from eligible studies: study characteristics (first author, year of publication, country, and study design), participant characteristics (sample size, age, and gender), type of OCT machine, and measured parameters. The primary outcomes were accuracy, sensitivity, specificity, and diagnostic odds ratio (DOR) of key OCT parameters distinguishing early glaucomatous eyes from healthy control eyes.

### Risk of Bias Assessment and Applicability

The methodological quality of each study was examined according to the Quality Assessment of Diagnostic Accuracy Studies-2 (QUADAS-2) tool.^[21]^ The seven ordinal QUADAS-2 items, which assess the quality of included studies based on the risk of bias and applicability concerns, were extracted for each study. Each study was assessed as having low, high, or unclear risk of bias (four domains) and applicability concerns (three domains). Two review authors (MH and AD) independently assessed the risk of bias according to the predefined criteria. Disagreements regarding item-specific and overall quality ratings were resolved by a third reviewer (ZK).

### Statistical Analysis

We performed a diagnostic NMA to explore diagnostic OCT parameters that can best identify early glaucoma. To evaluate the relative performance of OCT, we calculated pooled sensitivity and specificity for each diagnostic test compared with the reference standard of characteristic glaucomatous optic nerve damage ± glaucomatous VF defects.

To display the network map of studies reporting the diagnostic accuracy of key OCT parameters, we constructed a graph in which node size was proportional to the number of study participants, and line thickness represented the number of comparisons between tests. We calculated average sensitivity and specificity estimates, along with their derived relative values, and their corresponding 95% credible intervals (CrIs) using a bivariate random-effects NMA model.

In this study, accuracy was defined as the ability of each parameter to distinguish preperimetric or early glaucomatous eyes from healthy eyes. In almost all previous glaucoma diagnostic studies, the accuracy of average, superior, and inferior parameters was higher than that of nasal and temporal areas, a finding consistent across overall and subgroup analyses.^[16,17,18,19,20,21,22,23,24,25,26,27,28,29]^ Therefore, we excluded nasal and temporal parameters to enable comparison of more relevant measures. Damage to macular GCC/GCIPL layers seen on OCT was referred to as “macular parameters.” Heterogeneity was assessed using a forest plot. All analyses were performed using R (version 3.4.3; Comprehensive R Archive Network) and the packages rstan (version 2.17.3), loo (version 2.0.0), and plyr.

##  RESULTS

**Figure 1:**
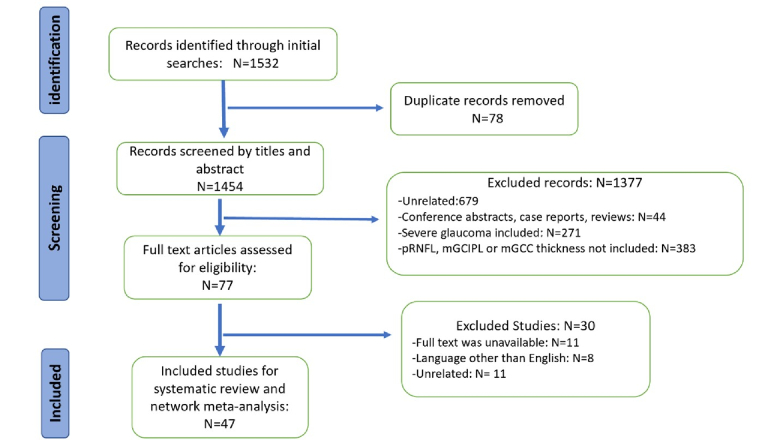
Preferred Reporting Items for Systematic reviews and Meta-Analyses (PRISMA) flowchart of the study selection process.

As shown in Figure 1, a total of 1532 studies were retrieved in our screening, of which 78 duplicates were removed, and 1377 articles were excluded by titles and abstracts. We further excluded 30 studies due to unavailable full-text versions, languages other than English, or the use of different diagnostic standards. Finally, according to our eligibility criteria, 47 studies were included in the systematic review.^[10,22,23,24,25,26,27,28,29,30,31,32,33,34,35,36,37,38,39,40,41,42,43,44,45,46,47,48,49,50,51,52,53,54,55,56,57,58,59,60,61,62,63,64,65,66,67]^ Most of the included studies were cross-sectional (80.8%), except for four (8.5%) case-control studies^[10,43,55,61]^ and five (10.6%) cohort studies.^[34,37,38,46,50]^ The total sample size comprised 12,723 eyes from 8177 participants. The flow diagram of the literature search is shown in Figure 1.

The included studies were published between January 2004 and March 2024 and spanned a diverse range of geographical regions. Of the 47 studies, the country-specific distribution was as follows: South Korea (10 studies, 21.3%), USA (9 studies, 19.1%), India (6 studies, 12.8%), Japan (5 studies, 10.6%), China (4 studies, 8.5%), Brazil (3 studies, 6.4%), and Turkey (2 studies, 4.3%). Additionally, one study each (2.1% per country) was conducted in Germany, Hungary, Taiwan, Malaysia, Singapore, the Philippines, Mexico, and Ethiopia.

The most commonly applied OCT machine was Cirrus (Carl Zeiss Meditec Inc., Dublin, CA, USA) in 17 studies (36.1%), followed by Spectralis (Heidelberg Engineering, Heidelberg, Germany) in 11 studies (23.4%), RTVue (Optovue Inc., Fremont, CA, USA) in 10 studies (21.3%), and Topcon (3D-OCT, Topcon, Tokyo, Japan) in 7 studies (14.8%). Two studies (4.2%) applied both Cirrus and Spectralis devices.

Different protocols were compared in terms of their performance in diagnosing glaucoma: 18 studies (38.2%) compared RNFL versus GCC; 8 studies (17%) compared RNFL versus ONH parameters such as BMO-MRW; and 11 studies (23.4%) compared ONH parameters versus GCC. Seven studies (14.8%) measured only RNFL parameters, and three studies (6.3%) measured only GCC parameters in early glaucoma. Detailed characteristics of the included studies are summarized in Table 1.

### Quality Assessment

**Figure 2:**
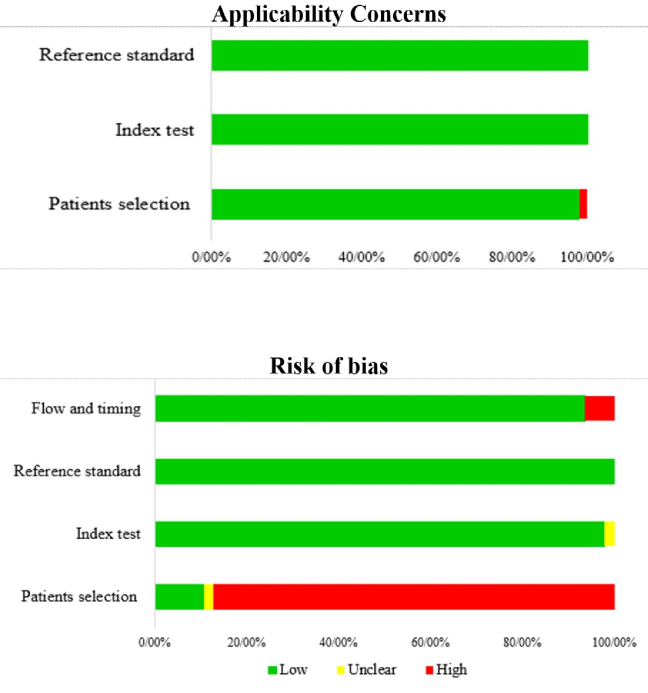
Risk of bias and applicability concerns graph on each QUADAS-2 domain presented as percentages across included studies.

The methodological quality of the 47 included studies was assessed using QUADAS-2 (detailed in Supplementary Material 3, summarized in Figure 2). A high risk of bias was found in the “patient selection” domain for 87.2% of studies, mainly due to cross-sectional or case-control designs that preselected participants based on known glaucoma status. Some concerns were also noted in the “reference standard” domain: although most studies used VF testing, a subset relied on optic disc appearance—an approach prone to subjectivity and variability. All studies showed low applicability concerns, indicating that their methods align well with real-world clinical practice.

### Results of NMA

We evaluated the diagnostic performance of three key OCT parameters in identifying early glaucoma. These parameters included peripapillary RNFL thickness, macular measurements, and ONH characteristics.

**Figure 3:**
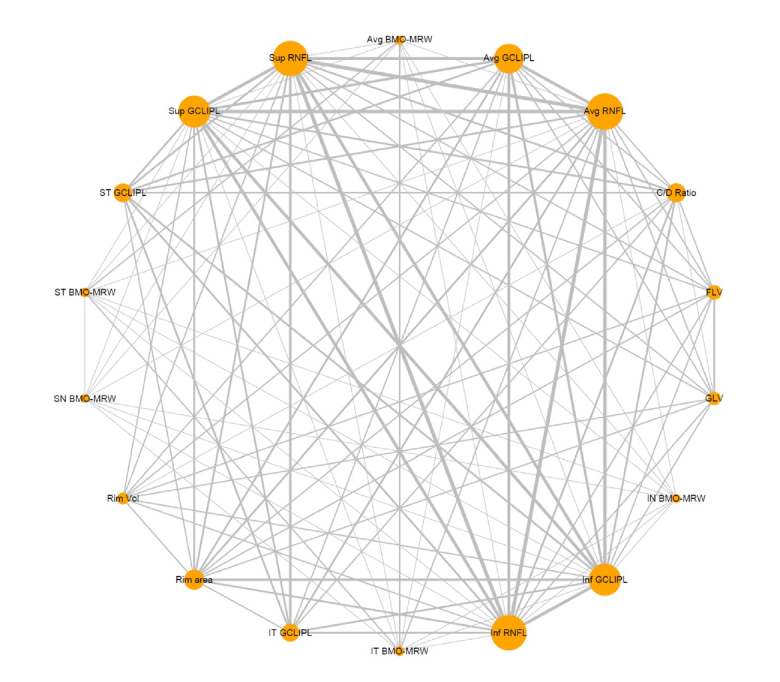
The network map of studies reporting the diagnostic accuracy of key OCT parameters. The circle (node) size is proportional to the number of study participants, and the thickness of lines between different OCT parameters represents the number of comparisons between the two tests.

To display the network, we constructed a graph where nodes represent different screening tests and edges represent head-to-head comparisons. As shown in Figure 3, RNFL and GCIPL are the parameters most frequently evaluated and compared in the included studies.

Our analysis revealed that the overall diagnostic accuracy was comparable across all three categories of parameters. The diagnostic accuracy of RNFL parameters ranged from 0.73 to 0.77 across different measurements. Among these, both average RNFL and inferior RNFL showed the highest accuracy at 0.77. Specifically, the inferior RNFL had a DOR of 13.57 (8.69-20.73), while the average RNFL exhibited a DOR of 12.37 (9.22-16.20).

**Table 1:** Characteristics of included studies

**Author/year**	**Location**	**Study design**	**Sample size (eyes)**	**Participants**	**Age (years ± SD)**	**Sex**	**OCT machine**	**Measured parameters**
Nouri-Mahdavi et al 2004^[10]^	USA	Observational case control	151	Mild glaucoma + control	66 ± 10.3	M = 50 F = 101	2000 OCT	pRNFL (average + sectors)
Leung et al 2009^[54]^	China	Cross-sectional	223	POAG + control	54 ± 14.6	Unknown	Cirrus	pRNFL (average + sectors)
Li et al 2009^[33]^	China	Cross-sectional	141	Mild to advanced glaucoma + control	51.05 ± 16.82	M = 81 F = 60	RTVue-100	pRNFL (average, sup, inf) CDR, disc area, rim area, cup area, rim volume
Garas et al 2010^[29]^	Hungary	Cross-sectional	286	OHTN + pre-perimetric + mild glaucoma + control	57.6 ± 11.7	M = 126 F = 160	RTVue-100	pRNFL (average + sectors) GCLIPL (average + sectors + FLV) CDR, rim area
Rao et al 2010^[55]^	USA	Case-control	214	POAG + control	68.3 ± 10.34	Unknown	RTVue-100	pRNFL (average + sectors) GCC (average + sectors + GLV + FLV) CDR, rim area, rim volume, disc area
Moreno et al 2011^[35]^	Brazil	Cross-sectional	123	Mild glaucoma + control	64.3 ± 11.8	M = 49 F = 74	RTVue-100	pRNFL (average + sectors) GCLIPL (average + sectors)
Nakatani et al 2011^[47]^	Japan	Cross-sectional	64	Pre-perimetric glaucoma + control	61.5 ± 7.7	M = 33 F = 31	Topcon	pRNFL (average + sectors) Macular thickness
Sung et al 2012^[52]^	South Korea	Cross-sectional	743	Pre-perimetric + mild glaucoma + control	53.1 ± 12.6	Male = 322 Female = 421	Cirrus	pRNFL (average, sectors) CDR, rim area, and volume
Wu et al 2012^[53]^	USA	Cross-sectional	146	POAG + control	69.2 ± 13	M = 60 F = 86	Spectralis	pRNFL (average + sectors)
Lisboa et al 2012^[50]^	USA	Observational cohort	134	Glaucoma suspect	66.1 ± 9.3	M = 52 F = 82	Spectralis	pRNFL (average, sectors) CDR, rim area, and volume
Akashi et al 2013^[49]^	Japan	Cross-sectional	232	POAG + control	48.3 ± 10.6	M = 102 F = 130	Cirrus + RTVue + Topcon	pRNFL (average + sectors) GCLIPL (average + sectors)
Lisboa et al 2013^[34]^	USA	Cohort	142	Glaucoma suspect	65.9 ± 9.1	M = 53 F = 89	RTVue-100	pRNFL (average + sectors) GCLIPL (average + sectors + GLV + FLV) CDR, rim area, rim volume, disc area, cup area
Rao et al 2013^[36]^	India	Cross-sectional	106	Pre-perimetric glaucoma + control	54 ± 10.6	Unknown	RTVue-100	pRNFL (average + sectors) GCLIPL (average + sectors + GLV + FLV) CDR, rim area, rim volume, disc area, cup area
Kanamori et al 2013^[40]^	Japan	Cross-sectional	179	Pre-perimetric glaucoma + mild glaucoma + control	49.9 ± 11.7	M = 80 F = 99	Topcon	pRNFL (average + sectors) GCLIPL (average + sectors)
Arintawati et al 2013^[42]^	Japan	Cross-sectional	261	Pre-perimetric glaucoma + mild to advanced glaucoma + control	58.9 ± 12.15	M = 112 F = 149	RTVue-100	pRNFL (average + sectors) GCC (average + sectors + GLV + FLV)
Nouri-Mahdavi et al 2013^[63]^	USA	Cross-sectional	150	POAG + control	66.1 ± 6.0	M = 56 F = 94	Cirrus	pRNFL (average + sectors) GCLIPL (average + sectors)
Sullivan-Mee et al 2013^[46]^	Mexico	Cross-sectional cohort	200	Mild glaucoma + control	68.9 ± 9.1	M = 190 F = 10	Spectralis	pRNFL (average+ sectors) macular thickness
Sung et al 2014^[45]^	South Korea	Cross-sectional	179	Pre-perimetric glaucoma + mild glaucoma + control	54.22 ± 12.7	M = 99 F = 80	Cirrus	pRNFL (average + sectors) GCLIPL (average + sectors)
Begum et al 2014^[41]^	India	Cross-sectional	136	Pre-perimetric glaucoma + perimetric glaucoma + control	47 ± 11	Unknown	Cirrus	pRNFL (average + sectors) GCLIPL (average + sectors) CDR, rim area, rim volume
Yamada et al 2014^[43]^	Japan	Retrospective case control	122	Pre-perimetric glaucoma + mild to advanced glaucoma + control	56.9 ± 14.7	M = 69 F = 53	Spectralis	pRNFL GCC
Hwang et al 2015^[57]^	South Korea	Cross-sectional	446	Pre-perimetric glaucoma + mild, moderate, advanced POAG + control	51.7 ± 13.8	Unknown	Cirrus	GCLIPL (average + sectors)
Yang et al 2015^[61]^	USA	Cross-sectional case-control	210	POAG + control	71.4 ± 10.2	M = 99 F = 111	Topcon	pRNFL (average + sectors)
Kim et al 2015^[59]^	South Korea	Cross-sectional	227	Pre-perimetric glaucoma + mild glaucoma + control	54.6 ± 11.8	M = 112 F = 115	Cirrus	pRNFL (average + sectors) GCLIPL (average + sectors)
Gmeiner et al 2016^[30]^	Germany	Cross-sectional	181	OHTN + pre-perimetric glaucoma + mild glaucoma + control	62.8 ± 10.2	M = 84 F = 97	Spectralis (GMPE)	pRNFL (average + sectors) BMO-MRW (average + sectors)
Gracitelli et al 2016^[31]^	Brazil	Cross-sectional	155	POAG + NTG + control	62.5 ± 13.8	M = 75 F = 80	RTVue-100	pRNFL (average + sectors) GCC (average + sectors)
Lee et al 2016^[51]^	South Korea	Prospective cross-sectional	120	POAG + control	59.9 ± 11.5	M = 53 F = 67	Spectralis + Topcon	pRNFL (average + sectors) GCLIPL (average + sectors)
Kim et al 2016^[60]^	South Korea	Cross-sectional	136	POAG + control	60 ± 9.8	M = 65 F = 71	Spectralis + SLO	pRNFL (average + sectors) GCLIPL (average + sectors) GCC
Aydoğan et al 2017^[24]^	Turkey	Cross-sectional	326	OHTN + pre-perimetric glaucoma + mild glaucoma + control	48.76 ± 7.12	M = 114 F = 212	RTVue-100	pRNFL (average + sectors) GCLIPL (average + sectors + GLV + FLV) CDR, rim area, rim volume
Lee et al 2017^[32]^	South Korea	Cross-sectional	184	Pre-perimetric glaucoma + mild glaucoma + control	58.23 ± 10.64	Unknown	Topcon	pRNFL (average + sectors) GCLIPL (average + sectors)
Xu et al 2017^[58]^	China	Cross-sectional	703	POAG + OHTN + glaucoma suspect + Control	46.6 ± 18.1	M = 352 F = 351	Cirrus	pRNFL (average + sectors) GCLIPL (average + sectors) CDR, rim area, rim volume
Kim et al 2017^[44]^	South Korea	Cross-sectional	132	Mild glaucoma + Control	59.2 ± 11.32	M = 43 F = 89	Spectralis	pRNFL (average + sectors) GCLIPL (average + sectors)
Dagdelen et al 2018^[62]^	Turkey	Cross-sectional	260	POAG + OHTN + control	59.31 ± 9.96	Unknown	Cirrus	pRNFL (average + sectors) CDR, rim area, rim volume, disc area
Deshpande et al 2019^[27]^	India	Retrospective cross-sectional	337	Pre-perimetric glaucoma + mild glaucoma + control	57.67 ± 9.37	M = 181 F = 156	Cirrus	pRNFL (average + sectors) GCLIPL (average + sectors)
Stagg et al 2019^[37]^	USA	Observational cohort	113	Glaucoma suspect	67.1 ± 9.9	M = 43 F = 70	Spectralis	pRNFL (average + sectors) BMO-MRW (average + sectors)
Abrol et al 2020^[22]^	India	Cross-sectional	1380	Pre-perimetric glaucoma + mild POAG + control	54.76 ± 7.95	M = 728 F = 652	Cirrus	pRNFL (average + sectors) GCLIPL (average + sectors)
Aquino et al 2020^[23]^	Philippines	Retrospective cross-sectional	96	Mild glaucoma + glaucoma suspect + control	61.55 ± 8.12	M = 46 F = 50	Cirrus	pRNFL (average + sectors) GCLIPL (average + sectors)
Chua et al 2020^[48]^	Singapore	Cross-sectional	846	Mild, moderate, advanced POAG + control	65 ± 9	M = 567 F = 279	Cirrus	pRNFL (average + sectors) GCLIPL (average + sectors)
Taia et al 2020^[38]^	Taiwan	Observational cohort	78	Pre-perimetric glaucoma + control	56.4 ± 11.1	Unknown	Cirrus	pRNFL (average + sectors) GCLIPL (average + sectors) CDR, rim area, rim volume, disc area
Deshpande et al 2021^[28]^	India	Retrospective cross-sectional	138	Pre-perimetric glaucoma + control	54.29 ± 10.42	M = 58 F = 80	Cirrus	pRNFL (average + sectors) GCLIPL (average + sectors) CDR, rim area
Bak et al 2022^[25]^	South Korea	Cross-sectional	196	Mild glaucoma + control	66.1 ± 12.6	Unknown	Cirrus	pRNFL (average + sectors) GCLIPL (average + sectors) CDR, rim area
Zangalli et al 2022^[67]^	Brazil	Cross-sectional	273	Mild and moderate glaucoma + control	64 ± 12.2	M = 109 F = 164	Spectralis	pRNFL (average + sectors) BMO-MRW (average + sectors)
Yusof et al 2022^[39]^	Malaysia	Cross-sectional	127	Pre-perimetric glaucoma + mild glaucoma + control	65 ± 5	M = 51 F = 76	Spectralis (GMPE)	pRNFL (average + sectors) GCLIPL (average + sectors) BMO-MRW (average, sectors)
Choe et al 2023^[26]^	South Korea	Cross-sectional	144	Pre-perimetric glaucoma + mild glaucoma + control	59.3 ± 13.3	M = 66 F = 78	Cirrus + Spectralis	pRNFL GCLIPL
Mahmoudinezhad et al 2023^[56]^	USA	Cross-sectional	260	POAG + glaucoma suspect + control	53.1 ± 13.8	M = 101 F = 149	Spectralis (GMPE)	GCLIPL
Wu et al 2023^[66]^	China	Cross-sectional	394	Mild glaucoma ( POAG, PACG, NTG) + glaucoma suspect + control	42-71	M = 175 F = 219	Spectralis (GMPE)	pRNFL GCLIPL BMO-MRW
Abera et al 2023^[64]^	Ethiopia	Cross-sectional	188	POAG + glaucoma suspect + control	58 ± 10	M = 98 F = 90	Cirrus	pRNFL (average + sectors) GCLIPL (average + sectors)
Yadav et al 2024^[65]^	India	Cross-sectional	60	Pre-perimetric glaucoma + control	36.97 ± 13.3	M = 33 F = 27	Topcon	pRNFL (average + sectors) GCLIPL (average + sectors) VCDR
pRNFL, peripapillary retinal nerve fiber layer; GCIPL, ganglion cell plus inner plexiform layer; GCC, ganglion cell complex; FLV, focal loss volume; GLV, global loss volume; CDR, cup to disc ratio; BMO-MRW, Bruch's membrane opening-minimum rim width; OHTN, ocular hypertension; POAG, primary open-angle glaucoma; PACG, primary angle closure glaucoma; NTG, normal tension glaucoma; PPG, pre-perimetric glaucoma.								

**Table 2:** Test performance against clinical diagnostic (perimetry and optic disc examination were used as reference standards)

**Test**	**Number of patients**	**Number of studies**	**Sensitivity**	**Specificity**	**Accuracy**	**Diagnostic odds ratio [rank]**
Inf RNFL ${}^{*,†}$	7269	35	0.70 (0.62, 0.78)	0.85 (0.80, 0.89)	0.77	13.57 (8.69 to 20.73)
Avg RNFL ${}^{*}$	9025	41	0.71 (0.65, 0.76)	0.83 (0.79, 0.87)	0.77	12.37 (9.22 to 16.20)
GLV	1049	5	0.70 (0.55, 0.81)	0.81 (0.70, 0.89)	0.76	11.67 (5 to 21.22)
C/D ratio ${}^{*}$	2488	10	0.70 (0.57, 0.81)	0.81 (0.74, 0.87)	0.75	10.76 (6.22 to 16.98)
IT GCLIPL	3015	13	0.68 (0.54, 0.79)	0.83 (0.76, 0.89)	0.75	11.11 (6.03 to 18.09)
Rim area	2899	11	0.67 (0.56, 0.76)	0.82 (0.75, 0.88)	0.75	10.24 (5.51 to 16.65)
ST GCLIPL	2387	11	0.68 (0.54, 0.81)	0.80 (0.72, 0.87)	0.74	9.64 (5.21 to 15.86)
Inf GCLIPL	6509	27	0.63 (0.53, 0.73)	0.84 (0.79, 0.88)	0.74	9.53 (6.43 to 13.48)
Avg GCLIPL	6805	29	0.63 (0.54, 0.72)	0.84 (0.79, 0.88)	0.74	9.32 (6.57 to 12.66)
Sup RNFL	7230	35	0.65 (0.57, 0.71)	0.82 (0.77, 0.86)	0.73	8.68 (6.72 to 10.95)
FLV	1049	5	0.62 (0.44, 0.79)	0.83 (0.74, 0.89)	0.72	8.91 (4.12 to 15.79)
ST BMO-MRW ${}^{†}$	694	4	0.58 (0.44, 0.70)	0.86 (0.75, 0.94)	0.72	10.56 (4.16 to 20.17)
Sup GCLIPL	6509	27	0.60 (0.49, 0.69)	0.83 (0.78, 0.88)	0.71	7.65 (5.28 to 10.56)
IT BMO-MRW	694	4	0.57 (0.40, 0.73)	0.85 (0.75, 0.92)	0.71	9.07 (3.45 to 18.43)
Avg BMO-MRW ${}^{†}$	694	4	0.56 (0.36, 0.75)	0.86 (0.75, 0.94)	0.71	10.41 (3.15 to 22.53)
IN BMO-MRW	694	4	0.50 (0.27, 0.71)	0.84 (0.71, 0.93)	0.67	6.33 (2.09 to 13.47)
SN BMO-MRW	694	4	0.49 (0.30, 0.68)	0.83 (0.70, 0.93)	0.66	5.76 (2.0 to 11.86)
Rim vol	823	4	0.54 (0.36, 0.72)	0.67 (0.50, 0.82)	0.60	2.95 (0.93 to 6.76)
95% CI, 95% confidence interval, ${}^{*}$ top three index tests with the highest sensitivity, ${}^{†}$ top three index tests with the highest specificity.						

**Figure 4:**
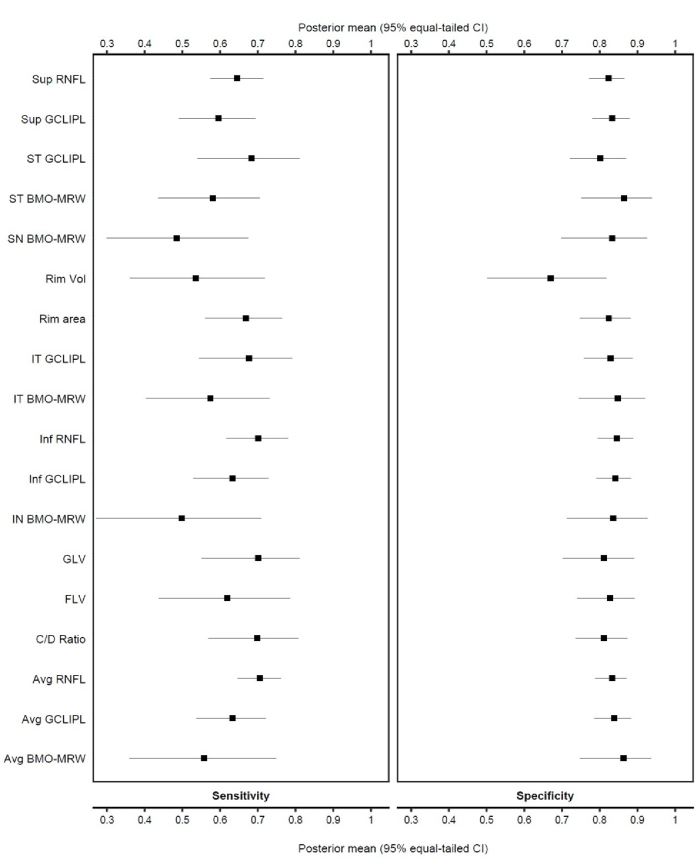
Forest plot showing the sensitivity and specificity of each OCT parameter in discriminating healthy participants from patients with early glaucoma.

For macular parameters, the inner retinal layers—comprising the macular nerve fiber layer, GCIPL, and GCC—were evaluated using various OCT platforms. Accuracies across macular metrics ranged from 0.71 to 0.76. Among these, GLV emerged as the most accurate macular parameter, with an accuracy of 0.76 (DOR: 11.67 [5-21.22]). This was closely followed by inferotemporal GCIPL, which showed an accuracy of 0.75 (DOR: 11.11 [6.03-18.09]) and average GCIPL demonstrating an accuracy of 0.74 (DOR: 9.32 [6.57-12.66]).

For ONH parameters, the pooled accuracies varied more substantially, ranging from 0.60 to 0.75. RA and cup-to-disc (C/D) ratio were the most accurate ONH parameters, each showing an accuracy of 0.75. RA had a DOR of 10.24 (5.51-16.65), while the C/D ratio demonstrated a DOR of 10.76 (6.22-16.98). The lowest diagnostic performance was observed for rim volume, with an accuracy of only 0.60 (DOR: 2.95 [0.93-6.76]).

Overall, the highest diagnostic accuracies for glaucoma detection were observed for average and inferior RNFL parameters, both achieving an accuracy of 0.77, and the macular GLV parameter with an accuracy of 0.76. These were followed by inferotemporal GCIPL and two ONH parameters— RA and C/D ratio—each with an accuracy of 0.75. Detailed performance characteristics of all evaluated parameters are provided in Table 2. To help illustrate heterogeneity across studies,^[68]^ the results are presented in a forest plot ordered by sensitivity and specificity [Figure 4].

##  DISCUSSION

Glaucoma is a slow, progressive degeneration of RGCs and their axons, resulting in a distinct appearance of the optic disc and retinal nerve fiber layer.^[69]^ When glaucoma is suspected during an ophthalmologic examination, diagnostic testing is needed to confirm damage. Standard automated perimetry (SAP) provides valuable information about functional damage; however, VF testing depends on patient compliance and cooperation, which can be challenging in clinical settings.^[70,71]^ Inter- and intra-subject variability also make it difficult to reliably detect small changes, especially in early glaucoma.^[72,73]^ Furthermore, structural damage is known to precede detectable functional loss; approximately 40% of axons must be lost before functional deterioration appears on SAP.^[74]^

One of the most commonly used imaging techniques is OCT, which provides several parameters revealing RNFL, macular and ONH damage caused by glaucoma. In this meta-analysis, we pooled data on the accuracy of several OCT parameters from eligible studies to determine which parameters better detect early glaucoma. Our results show highly similar accuracy among the most commonly used OCT protocols in discriminating healthy participants from patients with early glaucoma (0.77 accuracy for RNFL parameters, 0.76 for macular parameters, and 0.75 for ONH parameters).

Peripapillary RNFL remains the most frequently used structural parameter for glaucoma diagnosis. Our findings are consistent with the systematic review conducted by Oddone et al in 2016, which reported that average RNFL thickness assessed by RTVue OCT showed a sensitivity of 0.66 at a specificity of 0.95, outperforming macular parameters, although with only a small difference.^[75]^ Following the 2016 review by Oddone et al,^[75]^ macular parameter measurements have gained more attention, and several additional studies have been published. However, the current NMA indicates that overall findings have remained relatively consistent since 2016.

The number of studies incorporating ONH parameters alongside RNFL and macular parameters is relatively limited compared with investigations focused solely on the latter two. Heterogeneity in the number of included studies may affect direct comparisons when few studies are included. Nevertheless, the findings from these studies are promising. RA and C/D ratio, alongside other ONH parameters, have diagnostic accuracy comparable to that of macular parameters; however, they do not surpass that of RNFL parameters. Automatic detection of the optic nerve margin at the end of Bruch's membrane, rather than at the RPE termination, and the improved resolution of spectral domain technology may enhance the accuracy of optic disc demarcation. However, ONH metrics like RA and C/D ratio are not available on all OCT devices, which may limit generalizability across imaging platforms.

Despite the good diagnostic accuracy of all OCT parameters, it is important to emphasize that none of them can be perfect. Inferior RNFL, the best-performing parameter, achieved an accuracy of 0.77, a sensitivity of 70%, and a specificity of 85% in our NMA. Hence, a considerable number of glaucoma patients were not diagnosed using this parameter alone, suggesting that combining parameters may improve diagnostic accuracy.^[76,77]^ With computer-assisted diagnosis and AI, single-path data are being rapidly replaced by multimodal fusion imaging, which combines two or more parameters. In the near future, we may not have to choose among OCT parameters for glaucoma evaluation, as multimodal printouts providing data from three anatomical regions affected by glaucoma may become standard.

As digitalization progresses, integrating reflectance measurements of RNFL with its thickness may further augment diagnostic power. The ROTA (Retinal Nerve Fiber Layer Optical Texture Analysis) algorithm, which combines RNFL thickness and reflectance measurements from standard OCT scans, has the advantage of analyzing wide retinal areas, including the arcuate bundles, papillomacular bundle, and peripapillary NFL. It could help diagnose glaucoma at an early stage by detecting small RNFL defects involving the papillofoveal bundle.^[78,79]^ Interpretation of the ROTA algorithm is currently subjective, but results from ongoing studies evaluating its diagnostic effectiveness, along with standardization of glaucoma detection based on structural and functional changes integrated by AI, may help bridge the gap in early glaucoma detection.^[80]^

### Limitations

Some studies were excluded due to a lack of access to the full-text manuscript, despite exhaustive efforts (including contacting authors and institutional library services). Given the relatively small number of these studies and their limited influence in the literature, their exclusion is unlikely to have introduced significant bias or materially affected the pooled estimates.

We found that individual studies were inconsistent in their reporting of sensitivity and specificity, with some reporting sensitivity at particular specificity cutoffs and others reporting optimal cutoff values. This heterogeneity made meta-analysis more complex.

There are also wide variations in gold standards to define glaucoma, including reliance on SAP, optic disc appearance, elevated IOP, or a combination of these factors. While these reflect real-world clinical practice, they increase heterogeneity across results. In the present research, many included studies exhibited selection bias, largely attributable to their case-control design and the heterogeneity in reference standards used to establish the diagnosis of glaucoma. Although this design does not inherently compromise relative test accuracy in direct comparisons, it is likely to overestimate accuracy. Consequently, these case-control studies may have inflated the overall diagnostic accuracy of NMA metrics. However, by excluding results comparing moderate or advanced glaucoma with healthy participants, we avoided overestimation bias in situations in which OCT would add little diagnostic value.^[81]^

Although OCT provides quantitative data, its interpretation requires integration with other clinical information, such as VF testing and optic disc evaluation, underscoring the need for multimodal diagnostic approaches.

In summary, our NMA suggests that while no single parameter is perfect, average and inferior RNFL thickness, followed by macular GLV, inferotemporal GCIPL, ONH RA and C/D ratio, may serve as the most reliable OCT-based indicators for early glaucoma diagnosis. Incorporating these parameters into routine clinical practice could enhance early detection and monitoring of glaucoma, particularly when used in combination with functional assessments such as VF testing. Future studies should explore the integration of these structural biomarkers with AI–driven models to further improve diagnostic precision and support personalized patient management.

##  Financial Support and Sponsorship

None.

##  Conflicts of Interest

None.
